# Recent Advances in Hydrogels via Diels–Alder Crosslinking: Design and Applications

**DOI:** 10.3390/gels9020102

**Published:** 2023-01-24

**Authors:** Sofia M. Morozova

**Affiliations:** Center NTI “Digital Materials Science: New Materials and Substances”, N.E. Bauman Moscow State Technical University, 2nd Baumanskaya St. 5/1, Moscow 105005, Russia; sofiionova@yandex.ru; Tel.: +7-985-910-8502

**Keywords:** hydrogel, Diels–Alder reaction, biomedical application, biocomposites, self-healing

## Abstract

The Diels–Alder (DA) reaction is a promising tool for obtaining covalently crosslinked hydrogels due to its reaction bioorthogonality, the absence of by-products, and the application of mild conditions without a catalyst. The resulting hydrogels are in demand for use in various fields of materials science and biomedicine. While the dynamic nature of the cycloaddition of diene and dienophile has previously been used extensively for the fabrication of self-healing materials, it has only recently spread to the expansion of the functional properties of polymer gels for bioapplications. This review describes strategies and recent examples of obtaining hydrogels based on the DA reaction, demonstrating that the emerging functional properties go beyond self-healing. The types of classifications of hydrogels are listed, depending on the type of reaction and the nature of the components. Examples of obtaining hydrogels based on the normal and inverse electron-demand DA reaction, as well as the application of hydrogels for cell culture, drug delivery, injectable gels, and wound dressings, are considered. In conclusion, possible developmental directions are discussed, including the use of diene–dienophile pairs with a low temperature for the reversal of DA reaction, the modification of nanoparticles by diene and/or dienophile fragments, and new applications such as ink for 3D printing, sensing hydrogels, etc.

## 1. Introduction

**Figure 1:**
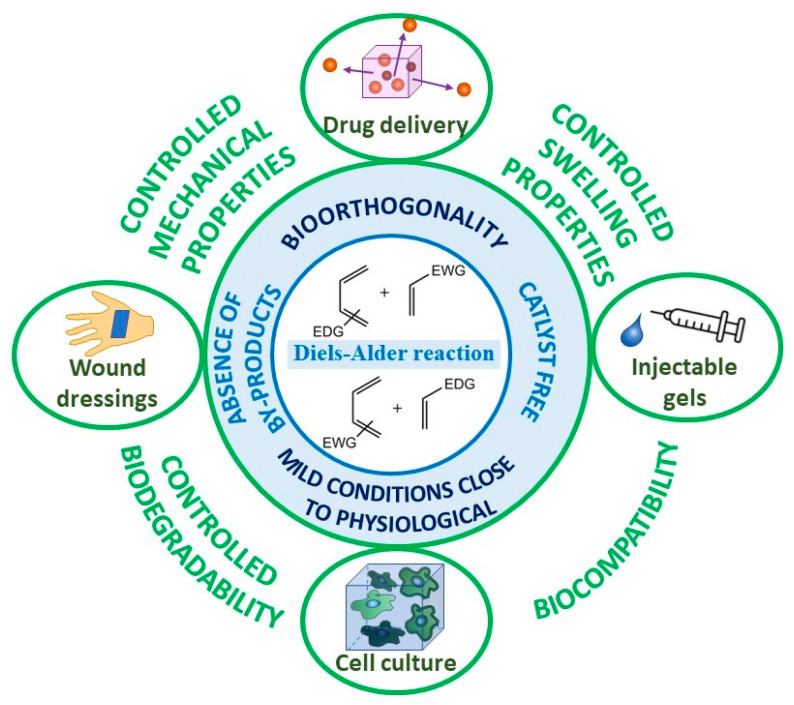
Illustration of various types of Diels–Alder-based hydrogels and their possible bioapplications.

The Diels–Alder reaction (DA) was discovered by German scientists Otto Diels and Kurt Alder in 1928 [1], for which they received the Nobel Prize in 1950. This reaction consists of the [2+4]-cycloaddition of compounds with a conjugated system of double bonds (diene component) with compounds having a double or triple bond (dienophilic component), leading to the formation of a six-membered cycle. Since its discovery, the DA reaction has gained increasing demand in various fields of chemistry, including organic synthesis for the production of cyclo-containing building blocks [2], and in macromolecular design for the production of gels, dendrimers, and brush-like polymers [3]; the thermal reversibility of the DA reaction led to the creation of a class of self-healing materials [4], and the speed and completeness of the interaction allowed the reaction to be used for the introduction of functional components (fluorescent dyes, proteins, physiologically active molecules) in various biomaterials [5]. Over the past 5 years, there has been an increased interest in the use of the DA reaction in biomaterials for the fabrication of biocompatible and biodegradable hydrogels [6,7]. However, existing reviews either considered the DA reaction only for self-healing applications [4], for bioapplications in general (not limited to gels) [6], or were aimed at synthesizing the initial components [7]. In connection with the active development of the research direction, it is interesting to summarize the results based specifically on DA hydrogels for bio-applications. Figure 1 illustrates the advantages of DA chemistry, both normal and inverse electron-demand, for biomaterials and their possible applications.

One of the advantages of this reaction is its bioorthogonality, i.e., its ability to occur inside living systems without interfering with natural biochemical processes. Moreover, the reaction could be proceeded under mild conditions (20–80 °C), without a catalyst and with the absence of by-products [8], which is especially important, for example, for the formation of cellular scaffolds, where by-products can be toxic to cells. Another important advantage is the high strength of the resulting hydrogels due to the formation of two covalent bonds per interacting pair of diene and dienophile. Physically/ionically crosslinking strategies usually lead to the formation of hydrogels with weak mechanical properties; therefore, in general, covalently crosslinked hydrogels have the advantage of exhibiting increased mechanical strength. Thus, by controlling the concentration of crosslinking, it is possible to change the mechanical properties of the hydrogel over a wide range to simulate certain living tissues [9]. The most common application of DA-based hydrogels involves the release of drugs [10,11,12,13], but the resulting gels are also in demand as scaffolds for cell cultures [14,15], injectable gels [16,17,18], and wound dressings [19].

This review is focused on the recent examples of DA-based hydrogels for biomedical applications. The first part of this paper describes the features of the DA reaction and the resulting classification of hydrogels crosslinked by DA chemistry, including the normal and inverse electron-demand DA. Then, strategies for introducing the fragments that could participate in DA reactions are described, and various applications of hydrogels in biomaterials and nanomedicine are discussed. The conclusion is devoted to the description of the future development of this research area.

## 2. Hydrogels via Diels–Alder Crosslinking

### 2.1. Classification

The Diels–Alder reaction exhibits the following features: (i) regioselectivity, resulting mainly in ortho-para isomers relative to the position of the functional groups in the diene–dienophile pair; (ii) partial stereospecificity and stereoselectivity, depending on the exact example; in principal, it is possible to obtain two stereospecific isomers, i.e., the presence of two endo- and exo-adducts, depending on the position of the most significant substituent (electron acceptor and/or conjugating group) relative to the diene π-system; (iii) thermal reversibility, i.e., reversible decomposition of the resulting 6-membered cycle back to diene and dienophile; and (iv) attributability of the reaction to normal or inverse electron-demand type, depending on the nature of the substituents (electron donor or electron acceptor) of diene and dienophile [1,2,3]. Since the focus of this review is on the gel materials, this work considers only the last feature of the reaction, while the first two have not yet shown a significant role in the application of hydrogels for biomedicine [7], and the third type is characterized by high temperatures of the reverse reaction (>100 °C) [20], which is not suitable for biological systems which are unstable at this temperature. Thus, the hydrogels in this review are classified primarily by the electronic type of reaction DA—normal or inverse.

**Figure 2:**
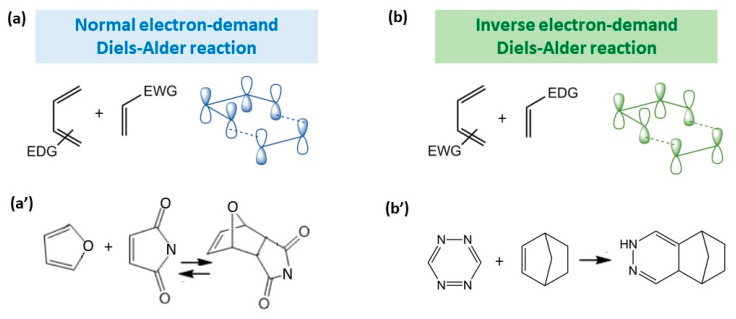
Illustration of the orbital interactions in normal (**a**) and inverse (**b**) electron-demand Diels–Alder reactions and the most common examples of these reactions for normal (**a′**) and inverse (**b′**) electron-demand Diels–Alder reactions.

Figure 2 illustrates the schematics of normal and inverse electron-demand DA, with examples of the reactions of real compounds.

The normal electron-demand Diels–Alder reaction involves electron-rich donating dienes (EDG group) and electronpoor withdrawing dienophiles (EWG group) owing to the matching of the diene’s highest occupied molecular orbital (HOMO) with the dienophile’s lowest unoccupied molecular orbital (LUMO) [21] (Figure 2a). The inverse electron-demand DA reaction was first discovered by Bachmann and Deno in 1949 [22], and it involves the opposite groups for diene and dienophile in comparison with normal electron-demand DA reaction, namely, the interaction of an dienophile with EDG group with an diene with EWG group. In frontier molecular orbital theory, this corresponds to the interaction of the LUMO of the dienophile with the HOMO of the diene [23] (Figure 2b). Examples of the most commonly used diene–dienophile functional pairs in modern polymer chemistry is the furan–maleimide coupling for normal electron-demand DA (Figure 2a′) and norbornene-tetrazine for inverse electron-demand DA (Figure 2b′).

**Figure 3:**
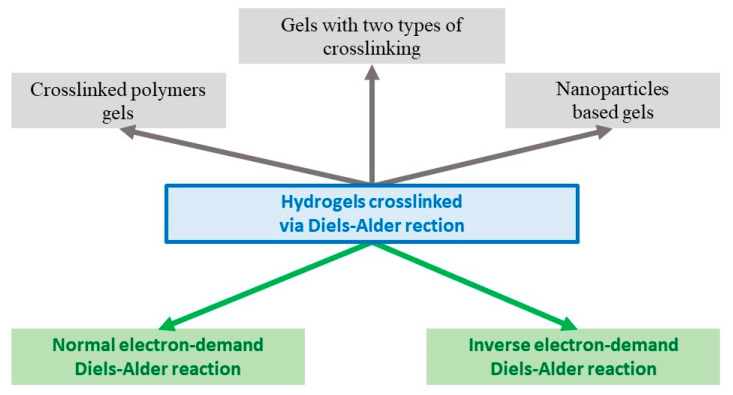
Classification of hydrogels obtained via Diels–Alder crosslinks.

In addition to the DA type of reaction, hydrogels are classified according to the type of components used for gelation (Figure 3).

A subclass of crosslinked polymers gels has been identified, which are either two crosslinked polymers [10], or a polymer crosslinked with a low molecular weight crosslinker [24]. Gels with two types of crosslinking are gels with interpenetrating polymer networks with crosslinks of a different nature; for example, when a strong and pH-resistant DA crosslinking is used in the gel with degradable crosslinking due to disulfide bonds [25]. Another distinguished subclass is the group called nanoparticle-based gels, in which the latter acts as a crosslinking agent using the DA reaction [26]. The following section provides examples of specific systems, according to the classification described above.

### 2.2. Hydrogel Design

#### 2.2.1. Normal Electron-Demand DA

The DA reaction of furan and maleimide is the most common, and it is studied in view of its simplicity and the accessibility of its initial components [3]. Thus, most of the examples of hydrogels reported in the literature describe the use of maleimide as the dienophile and furan as the diene. An additional advantage is that furan derivatives can be obtained from agricultural and forestry wastes; thus, furan-based chemistry has garnered recent attention due to its sustainability component [6]. Other pairs of dienophiles and dienes, for example, fulvene–maleimide [17], have been rarely used; however, the potentially application of other derivatives could lead to the kinetic control and the reversibility of the reaction.

*Crosslinked polymers gels:* hydrogels formed by the crosslinking of two polymer components, or a polymer and a low molecular weight crosslinker, are the most common types of DA-based gels [12,27,28].

Table 1 shows reported examples of diene and dienophile components used in hydrogel formation.

**Figure 4:**
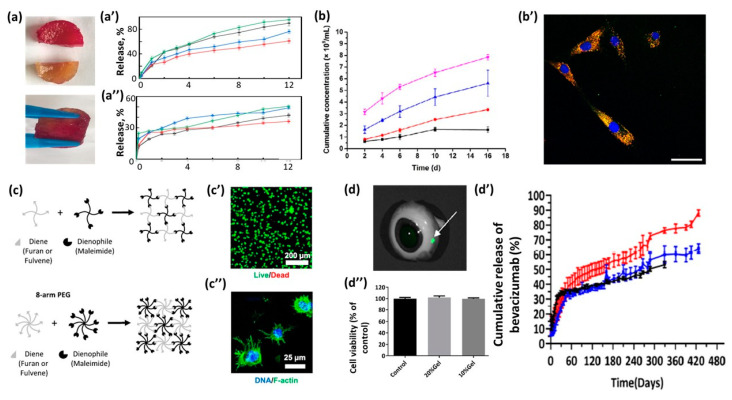
Application of DA-based hydrogels. (**a**) Self-healing property of sample hydrogel, top—cut hydrogel, and bottom—hydrogel healed for 5 h at 37 °C. (**a′**,**a″**). Cumulative release ratio of 5-fluorouracil from hydrogels with different diene/dienophile ratios under different simulated mediums: (**a′**) pH = 7.4, (**a″**) pH = 1.2; reproduced from ref. [12]. Copyright 2021, Elsevier Ltd. (**b**) Total iMSC-sEVs release ratio from the hydrogel after 16 days. Black line—no hyaluronidase; red line—0.1 μg/mL hyaluronidase; blue line—1 μg/mL hyaluronidase; and pink line—10 μg/mL hyaluronidase. (**b′**) Chondrocyte uptake of DiI (lipid membrane dye) and SYTO (RNA-specific dye) RNAselect-labeled iMSC-sEVs released from the hydrogel. The scale bar is 50 μm; reproduced from ref. [27]. Copyright 2021, Elsevier BV. (**c**) The 4-arm (top) and 8-arm (bottom) PEG hydrogels are prepared by mixing diene- (furan or fulvene) modified PEG with maleimide-modified PEG; (**c′**) viability of hMSCs encapsulated in DA hydrogel gels after 7 days, measured by a live/dead cytotoxicity assay; (**c″**) confocal fluorescence micrograph showing the spread of hMSCs cultured in DA hydrogels for 7 days. The actin cytoskeleton was stained with phalloidin (green), and the nuclei were stained with Hoechst (blue); reproduced with permission from ref. [15]. Copyright 2019, American Chemical Society. (**d**) Fluorescent image of a porcine eye 5 min after intravitreal injection of 4-arm PEG DA hydrogel at 37 °C (hydrogel marked by arrow); (**d′**) QMMUC-1 cell viability in the presence of hydrogel leachables from 10 and 20 wt% 4-arm PEG hydrogels after 24 h incubation; (**d″**) cumulative release of bevacizumab from 4-arm PEG DA hydrogel. Red, blue, and black lines correspond to 25, 20, and 10 wt% concentrations of hydrogels, respectively; reproduced from ref. [29]. Copyright 2022, American Chemical Society.

Bioapplications require biocompatibility and, in some cases, even biodegradability of the initial polymers, which limits the choice of the starting materials to the FDA approved substances [7]. Thus, the majority of examples described in the literature (Figure 4) are based on well-known biocompatible synthetic polymers such as poly(ethylene glycol) (PEG) [27], poly(glutamic acid) (PGA) [18], poly(caprolactone) [24], Jeffamine [10], or nature-derived polymers, such as gelatin [33], chitin [16], chitosan [28], and hyaluronan [19]. Some maleimide crosslinkers, such as maleimide-terminated 4-arm PEG, PEG dimaleimides, and bismaleimide are commercially available. However, the synthesis of furan, fulvene, and maleimide-modified polymers and nanoparticles (NPs) are in many cases, 1–2 step processes based on rather simple chemistry involving epoxy ring opening in furfuryl glycidyl ether by NH_2_ groups largely present in biopolymers [16,33], amide bond formation [27,29], Schiff’s base reaction, with the subsequent reduction of imine bonds [10], ester bond formation [11], or acetal formation [26].

*Gels with two types of crosslinking.* On the one hand, the resistance of the DA reaction to pH or catalytic decomposition can be considered as an advantage for creating strong and resistant gels; on the other hand, this could be a disadvantage if it is required to create a stimuli-responsive crosslinking. The strategy of creating gels that are different in chemical nature using two types of crosslinking can be used to create functional and stimuli-responsive gels [11,15,30,31,32] or to accelerate the formation of a gel to prevent excessive swelling [16], i.e., the introduction of pH-sensitive imine fragments [31,32] introduce to the gel a programmable sequential degradation under acidic environment and UV irradiation, which is beneficial for controlled drug release. A double cross-linked network hydrogel was prepared by combining a DA reaction and the coordination of catechol fragments with iron ions [30]. This hydrogel showed anti-EDTA performance and self-healing properties due to its supramolecular Fe^3+^-catechol bonds. An interesting example was the combination of both normal and inverse electron-demand DA reactions in the same hydrogel [15]. The first type of crosslink was responsible for the mechanical framework of the gel, and the second one, which was based on a faster reaction, was used for the introduction of a fluorescent dye.

*Nanoparticle-based gels.* Recently, there has been an increased interest in gels based on nanoparticles, including those covalently crosslinked with the gel [37]. Currently, there are only a few examples of hydrogels crosslinked with NPs via the DA reaction [26,34,35,36], and this is due to the greater complexity of the modification and characterization of NPs compared to linear polymers or low molecular weight compounds. Inorganic NPs could be modified by reacting with a dopamine-maleimide linker [35,36]. By applying this strategy, hydrogels based on the DA reaction of benzotriazole maleimide (BTM) functionalized Ag NPs and furan-containing gelatin were reported [36]. The incorporation of Ag NPs as cross-linkers led to an increase in the storage modulus of the gel, and a decrease in the swelling ratio in comparison to the NPs-free control. The obtained hydrogel also demonstrated improved cell viability (L-929 murine fibroblast cells) and enhanced drug release, which opens a new route to a number of potential biomedical applications, such as controlled therapeutic delivery or tissue engineering. By using a similar strategy, DA hydrogels formed from dopamine-maleimide modified TiO_2_ NPs and furan-modified gelatin were reported [35]. The use of nanocrystalline cellulose (CNC) nanoparticles in hydrogel formation is highly promising, due to their natural origin—sourced from natural wood, their biocompatibility, and their anisotropic rod shape [38]. The modification of CNC could be performed by ester formation with 3-maleimidopropionic acid [34], acetal formation with furfural [26], or by carbamation with isocyanate-modified furan or maleimide derivatives [39]. However, only the first two strategies were used to obtain DA cross-linked hydrogels [26,34]. At the same time, the authors used a large excess of polymer to CNC (>10), while an increase in the amount of CNC leads to the fibrillar structure of the gel [40], which plays an important role in cell growth [41]. Moreover, the biocompatibility of the gels remained undetermined.

#### 2.2.2. Inverse Electron-Demand DA

One of the disadvantages of a normal reaction DA crosslinking is the slow gelation time (several hours), due to which the gels undergo significant swelling [14]. One of the solution is to switch to a faster inverse electron-demand DA reaction [25]. However, the disadvantage of this reaction is the specificity of the compounds used and their lower availability compared to the normal electron-demand DA reaction. Few examples are known, and all of them are based on tetrazine–norbornene interaction [13,15,25]. This “click” reaction is carrying out without a catalyst, and produces only negligible quantities of nitrogen gas, without any no other toxic side products, making it very promising for bioapplications. Moreover, the nitrogen release could positively affect the formation of porous structures inside the hydrogel networks [13].

*Crosslinked polymers gels.* In a recent work [14], hydrogels formed by the crosslinking of two types of hyaluronan components, where one was modified by 4-(4,6-dimethoxy-1,3,5-triazin-2-yl)-4-methylmorpholinium chloride, and the other with 5-norbornene-2-methylamine through the formation of amide bonds. Tunable gelation, with gelation times from 4.4 min to 46.2 min, were achieved by tuning the composition and molar mass of the initial HA. The obtained gels were transparent, mechanically strong (Young modulus up to 1000 Pa), biodegradable, and cytocompatible, making them promising for 3D cell culture and imaging.

*Gels with two types of crosslinking.* This gel contains two types of crosslinks—an irreversible DA-based gel and a multi-stimuli diselenide crosslink gel were reported [13]. The hyaluronan polymer was modified, as described previously, with 5-norbornene-2-methylamine [14], and was crosslinked with a novel diselenide-ditetrazine cross-linker. By varying the polymer/crosslinker ratios from 1 to 4, gelation times increased from 155 to 509 s. Similarly, the mechanical strength of the hydrogel decreased by decreasing the molar ratio of the cross-linker from 3000 to 750 dyne/cm^2^. Due to the presence of the diselenide bond, the hydrogels possessed a stimuli-responsive drug release related to the degradation of the S–Se bond under the presence of 4-dithiothreitol or H_2_O_2_, as well as under near-infrared (NIR) irradiation, making them promising for use in photothermal therapy for tumor treatment. A similar approach was realized in methylcellulose- (MC) based gels with disulfide and DA crosslinks [25]. One component was based on MC modified with a carboxylic group, which then was reacted with 5-norbornene-2-methylamine, and the other component was based on MC-methylphenyltetrazine containing disulfide bonds. The obtained gels have a gelation time of <15 min at a physiological temperature and pH, and possess a Young’s modulus similar to that of brain tissue (1–3 kPa). The disulfide bonds in the hydrogel were degradable in the presence of thiols (which are naturally occurring in the biological environment) and demonstrated the ability to release proteins and chondroitinase ABC.

### 2.3. Application

This section will highlight examples of biomedical applications of DA-based hydrogels. According to the results published over the past 5 years, DA hydrogels have found the most widespread use as systems for drug delivery and as cellular scaffolds. Applications related to the injectability property are highlighted due to their potential connection with 3D printing, although they can be classified according to the first two points. Single application examples are described in Section 2.3.4.

#### 2.3.1. Drug Delivery

Hydrogels are already widely used in modern medicine as drug delivery systems. Due to the ability to control physical properties such as swelling, mesh size, and biodegradation, the release profile of the drug can be controlled using hydrogels. Moreover, hydrogels are able to protect labile drugs from decomposition, as well as to carry out a programmable (i.e., on demand, stimuli-responsive) release due to physicochemical interactions with encapsulated drugs [42].

Chitosan-based hydrogels are often used as carriers for drug delivery due to their ability to regulate swelling, and as well as drug release, by changing the pH of the medium. For example, hydrogels based on bismaleimide-crosslinked furan-modified chitosan demonstrated the pH-mediated release of chloramphenicol [10]. The total antibiotic release was achieved after 5 h in pH conditions which simulated intestinal fluid.

Introducing hydrogel self-healing properties could protect loaded drugs from being destroyed before arriving at the target [12]. Pectin/chitosan hydrogel crosslinked via DA reaction demonstrated self-healing properties due to the electrostatic interaction between the pectin (carboxylic group) and the chitosan (amino group). Pieces of hydrogel were cut and then healed for 5 h at 37 °C (Figure 4a), demonstrating the full recovery of its mechanical properties (the ability to bear a 500 g weight without being damaged).

The obtained hydrogel demonstrated cytocompatibility with fibroblast L929 cells and pH-controlled release of the model drug, 5-Fluorouracil, which was decreased at a pH of 1.2 and increased as the pH value rose to 7.4 (Figure 4a′,a″).

Hydrogels could be used for the release of cells. Diels–Alder crosslinked hyaluronic acid/PEG hydrogel was reported as an intraarticular delivery platform for hydrogel, as it could achieve the sustained release of mesenchymal stem cell-derived small extracellular vesicles (MSC-sEVs), mainly by degradation control [27]. The release of encapsulated iMSC-sEVs from hydrogels revealed the nearly complete absence of cell sedimentation (Figure 4b). The 2D images in Figure 4b′ show the efficiency of the uptake of iMSC-sEVs (Figure 4b′). An in vivo experiment revealed that the obtained hydrogel could enhance the efficacy of MSC-sEVs for osteoarthritis improvement.

#### 2.3.2. Cell Culture

One of significant limitations in the application of DA-based hydrogels as 3D cell culture scaffolds is the degradation of maleimide–furan gels under physiological conditions. Another limitation is related to the slow reaction rate of the DA reaction when using a furan–maleimide pair, which can lead to cell sedimentation. A possible solution may be associated with the use of a faster pair of DA reactions, for example, the inverse electron-demand DA reaction [14] or the normal electron-demand DA reaction, substituting the for the fulvene–maleimide pair [15], or the introduction of a second type of crosslinking to form a hydrogel (described in the Section 2.3.3). When the degradation of the DA adducts occurs in aqueous media, the released maleimides groups are susceptible to hydrolysis and can degrade to a maleamic acid that does not participate in the DA reaction.

To overcome these listed limitations, hydrogels based on the inverse electron-demand DA reaction, with a gelation time from 4.4 to 46.2 min, were designed [14]. It is shown that the rate of biodegradation of the obtained gels varied from hours to days, depending on the concentration of the enzyme and the amount of crosslinking. Pregel solutions possess a pink color due to the presence of methylphenyltetrazine; however, the color disappeared within hours, resulting in clear transparent hydrogels with a refractive index (RI) of 1.335, which is close to the RI of water. The obtained gels were fully transparent (transmittance > 90%) and biocompatible with the retinal cells, making this gel promising for cell 3D culture and imaging.

Hydrogels based on fulvene–maleimide has several advantages, such as a reaction time up to 10 times faster than other commonly used DA reaction pairs and stability maintained for months under physiological conditions [15]. The hydrogels were formed by 4- or 8-arm PEG, modified with fulvene or furan and maleimide (Figure 4c). It was demonstrated that the gelation time for 4-arm PEG fulvene–maleimide (20 min) was much faster than for the furan–maleimide pair (10 h). The obtained gels are promising in 3D cell culture platforms due to the high viability (89.3%) of mesenchymal stromal cells (hMSCs) after 7 days (Figure 4c′), as well as their good cell adhesion (Figure 4c″).

#### 2.3.3. Injectable Gels

Injectable hydrogels could flow under shear stress (for example, originating from pressing the gel through a syringe), resuming their well-defined shape after removal of the stress. Such behavior could be achieved by using (i) physically crosslinked gels [43], which possess shear-thinning properties, (ii) thermosensitive gels [44] or gels with dynamic bonds [45], which could liquified under external stimuli, or (iii) systems with gelation times in the range of tens of seconds to several minutes, allowing them to flow smoothly and then regain their shape due to fast gelation [46]. DA hydrogels are promising for the third strategy due to the ease of variation of the gelation time by adjusting the crosslinking density and temperature parameters.

A hydrogel with two types of crosslinking—physical hydrogen bonding and covalent DA bonds—has been used as an injectable gel for 3D cell growth and regenerative medicine [16]. The physical gel was initially formed, providing injectability, while the slow formation of the covalent DA crosslinking delivered the stability of the hydrogel over time, under physiological conditions. HeLa and mouse macrophage RAW264.7 cells encapsulated in hydrogel demonstrated stable proliferation and good self-assembly in multicellular spheroids. An in vivo study showed favorable injectability, in situ thermohelination, and good biocompatibility.

An injectable hydrogel created via the DA reaction between fulvene and maleimide-functionalized PEG, with a sustained release of T-cells through gradual hydrolysis, was reported [17]. It was shown that the variation of the molecular weight of the PEG used could change the gelation time in the range of 15 to 150 min, Young’s modulus in the range of 5 to 179 kPa, and the degradation time in the range of 7 to 114 h. The gel (5 wt%) could be injected through a narrow (26 G) needle and released encapsulated CD3+ T-cells with a linear release profile of up to 7 days.

An injectable hydrogel with prolonged release and improved resistance to degradation has been developed for the treatment of retinal diseases [29]. The hydrogel was formed in 5 min by the DA reaction of maleimide-terminated 4-arm PEG and furan-modified hyaluronic acid and was easily injected into an ex vivo porcine eye at 37 °C using a small needle (29 G). At 5 min post-injection, the gel (labeled with Fluor 750 C5) was observed in the vitreous at the injection site as a green dot (Figure 4d). The hydrogel showed high biocompatibility (Figure 4d′) and the sustained release of bioactive bevacizumab > 400 days (Figure 4d″).

#### 2.3.4. Other Applications

The self-healing hydrogel was obtained by the reaction of chitosan modified with furan and catechol fragments, dimaleimide PEG, and iron ions [30]. At the same time, the self-healing properties were originated not from DA crosslinking, but from the coordination bonds of iron ions with catechol. Moreover, the gel was also pH sensitive and demonstrated liquification at pH 3, as well as a color change from brown to black with a pH change from 5 to 8. The gel storage modulus after the self-healing process did not change significantly, and the cross-linking density and mechanical properties of the DN hydrogel can be controlled by varying the Fe^3+^-catechol ratio and the pH value, which makes this gel promising for implants and wound healing applications.

A hydrogel for wound dressings was fabricated by the formation of a semi-interpenetrating polymer network (semi-IPN) between DA crosslinked furan modified hyaluronic acid/4-arm PEG maleimide and halogen-free imidazolium poly(ionic liquids) (PIL) [19]. DA crosslinks were responsible for the gel mechanical properties, grafted deferoxamine (DFO) promoted angiogenesis, and PIL introduced antibacterial properties into the hydrogel. In the early stage (2 h), hydrogels eradiated the bacteria by the contact-killing model as a result of the highly effective antibacterial PIL. As the hydrogel further degraded (20 h), HA and DFO accelerated cell proliferation and promoted angiogenesis. The efficiency of wound healing was confirmed by in vitro and in vivo studies.

## 3. Conclusions

The DA reaction of cycloaddition is extremely promising for obtaining hydrogels with variable mechanical and swelling properties for biomedical applications. This reaction is attributed to “click” chemistry [8] and proceeds under biocompatible, mild experimental conditions, and the functional groups involved, diene and dienophile, are absent in natural biopolymers. Moreover, one of the most widely used components for normal electron-demand DA, furan, can be obtained from bio-renewable sources [47], which suggests economic benefits, as well as incorporation of green chemistry principles. Interestingly, the kinetics of crosslinking via DA can be regulate, providing fast or slow gelation, thereby expanding the range of biomedical applications. The use of nanoparticles as one of the components of the gel also contributes to the expansion of the functionality of the materials obtained. However, the examples currently published do not yet fully reveal the potential of DA crosslinks, and they are limited to several dienes and dienophiles—furan–maleimide and fulvene–maleimide for normal electron-demand DA, and tetrazine–norbornene for inverse electron-demand DA. Further development of DA hydrogels could be associated with several directions:

(i) Obtaining diene–dienophilic pairs capable of reacting reversibly under mild (biologically relevant) conditions (below 45 °C). For a standard furan–maleimide pair, the reversible DA reaction occurs above 100 °C [20], depending on the functional groups, which is suitable for self-healing materials, but too high for biological systems that break down above 45 °C [48], allowing for the expansion of biomedical applications by imparting self-healing properties; existing examples approached the temperature of the reverse DA in 50–60 °C [49,50]; however, the reaction occurred in non-aqueous media.
(ii) Novel applications of DA-based hydrogels, for example, the use of DA hydrogels as the basis of inks for 3D bioprinting (i.e., the presence of cells in the ink), which is possible due to the biorthogonality of the DA reaction and is promising for regenerative medicine and tissue engineering; existing works have already shown the possibility of creating injectable gels [16,17,29], including those containing cells [17], but full-fledged 3D objects, such as implants or tissues, have not yet been tested. Another prospective direction is the combination of DA hydrogels with microfluidic technologies to create organs on a chip.
(iii) The development of methods for modifying nanoparticles with fragments capable of participating in the DA reaction to expand the functionality of gels due to the task-specific properties of nanoparticles. For example, obtaining gels with fibrillar structure by using anisotropic particles is beneficial for recapturing the mechanical and permeable properties of the extracellular matrix [51]. The introduction of optically active DA fragments [52] or NPs [53,54], such as quantum dots, upconverting NPs, or plasmonic NPs, could be beneficial for the fabrication of hydrogels with sensing properties. Most of the published works are related to the physical interaction of particles with hydrogel [53], while covalent crosslinking, in particular DA, could avoid toxicity problems due to the strength of the covalent bonding of NPs with hydrogels and the improved visualization properties.

Finding solutions to the above issues will greatly expand the application range of hydrogels crosslinked via the Diels–Alder reaction.

## Figures and Tables

**Table 1:** Components for DA-based hydrogels. Diene (a) and dienophile (b) components, which are commonly used for hydrogel formation.

(a) Diene Component		(b) Dienophile Component	
Description	Reference	Description	Reference
Furyl modified Chitin	[16]	4-arm maleimide-terminated PEG and PEG dimaleimide	[15,16,17,18,19,26,27,29,30,31,32]
Furyl modified hyaluronan	[19,27,29,32]	Maleimide-terminated Jeffamine	[10,33]
Furyl modified hydroxypropylcellulose	[11]	Maleimide-modified cyclodextrin	[11,28]
Furyl modified poly(glutamic acid)	[18]	Bismaleimide	[13]
Furyl modified poly(caprolactone)	[24]	Maleimide-modified cellulose nanocrystals	[34]
Furyl modified gelatin	[33,34,35,36]	Maleimide-modified Ag NPs	[36]
Furyl modified cellulose nanocrystals	[26]	Maleimide-modified TiO_2_ NPs	[35]
Fulven modified PEG	[17]		

## Data Availability

Not applicable.
